# Hearing Asymmetry Biases Spatial Hearing in Bimodal Cochlear-Implant Users Despite Bilateral Low-Frequency Hearing Preservation

**DOI:** 10.1177/23312165221143907

**Published:** 2023-01-05

**Authors:** Snandan Sharma, Lucas H.M. Mens, Ad F.M. Snik, A. John van Opstal, Marc M. van Wanrooij

**Affiliations:** 1 Department of Biophysics, 6029 Radboud University, 198328 Donders Institute for Brain, Cognition and Behavior, Nijmegen, The Netherlands; 2 Department of Otorhinolaryngology, 6034 Radboud University Medical Centre, 198328 Donders Institute for Brain, Cognition and Behavior, Nijmegen, The Netherlands

**Keywords:** sound localization, human, psychophysics, hearing aid, electro-acoustic device, binaural, monaural, interaural level difference, interaural time difference

## Abstract

Many cochlear implant users with binaural residual (acoustic) hearing benefit from combining electric and acoustic stimulation (EAS) in the implanted ear with acoustic amplification in the other. These bimodal EAS listeners can potentially use low-frequency binaural cues to localize sounds. However, their hearing is generally asymmetric for mid- and high-frequency sounds, perturbing or even abolishing binaural cues. Here, we investigated the effect of a frequency-dependent binaural asymmetry in hearing thresholds on sound localization by seven bimodal EAS listeners. Frequency dependence was probed by presenting sounds with power in low-, mid-, high-, or mid-to-high-frequency bands. Frequency-dependent hearing asymmetry was present in the bimodal EAS listening condition (when using both devices) but was also induced by independently switching devices on or off. Using both devices, hearing was near symmetric for low frequencies, asymmetric for mid frequencies with better hearing thresholds in the implanted ear, and monaural for high frequencies with no hearing in the non-implanted ear. Results show that sound-localization performance was poor in general. Typically, localization was strongly biased toward the better hearing ear. We observed that hearing asymmetry was a good predictor for these biases. Notably, even when hearing was symmetric a preferential bias toward the ear using the hearing aid was revealed. We discuss how frequency dependence of any hearing asymmetry may lead to binaural cues that are spatially inconsistent as the spectrum of a sound changes. We speculate that this inconsistency may prevent accurate sound-localization even after long-term exposure to the hearing asymmetry.

## Introduction

Criteria for cochlear implantation have been expanded to allow adults with good low-frequency hearing thresholds and severe-to-profound high-frequency hearing loss in both ears to receive a single cochlear implant. The combined electric and acoustic stimulation (EAS) in the implanted ear and acoustic stimulation in the other provides these cochlear implant recipients with binaural low-frequency acoustic hearing, which has been shown to improve horizontal-plane sound localization (Dunn et al., 2010; Gifford et al., 2014; Plant & Babic; 2016; Kortje et al., 2020). However, these EAS listeners effectively have monaural (electrical) hearing for high frequencies and asymmetric (bimodal) hearing for mid frequencies. It remains poorly understood how these frequency-dependent hearing asymmetries affect sound-localization performance.

**Figure 1.**
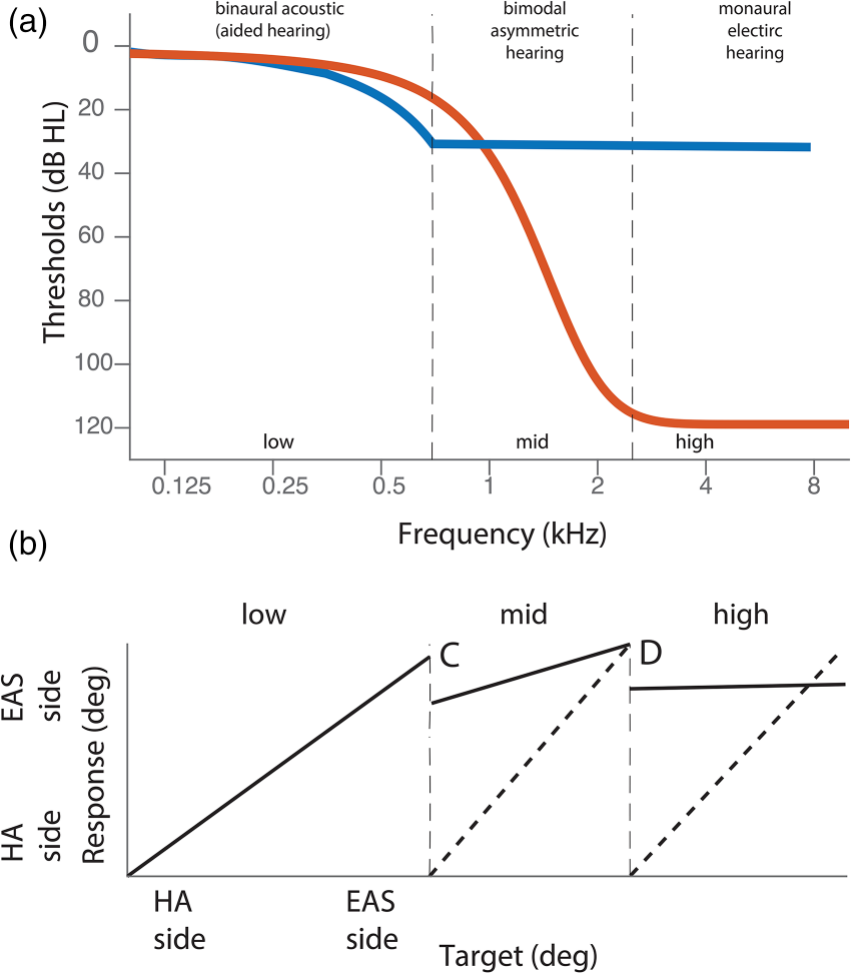
**Rationale on how hearing asymmetry in bimodal EAS users influences sound localization.** (a) Example hearing thresholds for a typical bimodal EAS user. The red line indicates hearing-aid ear, the blue line indicates the EAS ear. (b)–(d) Predicted sound localization performance if hearing asymmetries are not compensated for. (b) Low-frequency sounds can be accurately localized due to good binaural acoustic hearing thresholds. (c) Hearing asymmetry for mid-frequency sounds may introduce a localization bias toward the better-hearing EAS ear. (d) High-frequency sounds are likely to be perceived only in the EAS ear. Dashed diagonal lines indicate an ideal target response behavior. Solid black lines indicate predicted response to sounds with different frequency information. A bias is indicated by a shift of this line. HA – hearing aid, EAS – electro-acoustic stimulation.

The EAS listeners with low-frequency binaural hearing (Figure 1a, low frequency region) can potentially localize sounds in the horizontal plane using interaural time differences (ITDs; Figure 1b (Dorman et al., 2013; Gifford et al., 2013; Gifford Graham et. al., 2014 ; Dorman et al., 2016; Gifford & Stecker, 2020; Körtje et al., 2020). However, typical EAS listeners will have no high-frequency hearing in the non-implanted ear (Figure 1a, high frequencies) and so they lack access to interaural level differences (ILDs) necessary for high-frequency horizontal (Blauert, 1996) sound localization (Figure 1d). Importantly, for mid-frequency sounds, (Figure 1a, mid) current conventional electrical stimulation will not provide the temporal fine structure necessary for low-frequency ITD processing and the magnitude of low-frequency ILDs will be small (Macaulay et al., 2010; Figure 1c). Moreover, in this frequency range, acoustic hearing in the non-implanted ear is worse than electrical hearing in the implanted ear (Figure 1a, mid), introducing a hearing asymmetry that may make any mid-frequency binaural cues useless. Taken together, access to binaural cues for bimodal EAS listeners depends highly on the spectrum of the sound and likely depends on individual differences in hearing thresholds and hearing asymmetry across the ears.

In this study we investigate how the frequency-dependent binaural hearing asymmetries affect sound localization by bimodal EAS users. To that end, we present low-, mid-, mid-to-high, and high-frequency sounds and acutely introduce larger hearing asymmetries by turning on and off the devices. We quantify hearing asymmetry as a binaural difference in hearing thresholds separately for each participant, sound, and listening condition. We hypothesize that hearing asymmetries introduce localization biases to the better hearing ear (Figure 1b to d). This would suggest that idiosyncratic localization abilities as demonstrated in earlier studies (Dunn et al., 2010; Gifford Grantham et al., 2014; Dorman et al., 2016; Körtje et al., 2020) with errors ranging from near-normal (10 deg) to extremely poor (70 deg) may well depend on individual hearing asymmetries. Indeed, more symmetric hearing in bimodal EAS users correlates with the ability to process ITDs (Gifford Grantham et al., 2014) and localize sounds (Loiselle et al., 2015).

Furthermore, we hypothesize that, as hearing asymmetry depends on frequency (Figure 1a), sound localization by EAS listeners depends on the sound spectrum as well. Specifically, since bimodal EAS users with binaurally symmetric low-frequency hearing can rely on low-frequency ITDs (Gifford Grantham et al., 2014; Kortje et. al., 2020; Figure 1a), they should be able to localize low-frequency sounds a­ccurately (Figure 1b). Even without perfect hearing symmetry (e.g., if input levels are not equalized across ears), localization might still be unbiased, as ITD sensitivity might remain robust (Gifford Grantham et al., 2014; van Ginkel et al., 2019; Gifford & Stecker, 2020). In contrast, high-frequency sounds may be localized toward the implanted side (Figure 1d) due to a complete absence of audibility in the hearing-aid ear (Figure 1a) leading to an insurmountable asymmetry. For mid-frequency sounds the implant does not provide temporal fine structure information and hearing loss in the hearing-aid ear is often substantial (exceeding 30 dB HL in Figure 1a). In this case, bimodal EAS users might be able to utilize low-frequency ILDs (as suggested for bimodal users: Veugen et al., 2016; Sharma et al., 2021; and for bimodal EAS users: Gifford & Stecker 2020). If the hearing asymmetry (favoring the implanted ear) is not accounted for, this might yield a bias toward the implanted ear (Figure 1c). For mid-to-high frequency sounds, this asymmetry might be even more extreme, and sound localization could be biased anywhere from intermediate (Figure 1c) to extreme (Figure 1d).

## Methods

### Participants

Seven bimodal cochlear implant users (5 female, mean age 61 years; range 54–76 years) took part in the study, all having a severe sensorineural high-frequency hearing loss and low-frequency acoustic hearing in both ears. The participants had 6 months to 10 years of experience with an Advanced Bionics (California, USA; *n* = 2), Cochlear (Sydney, Australia; *n* = 3), or Med-El EAS (Innsbruck Austria; *n* = 2) and all had worn a contralateral hearing aid for at least 2 years up to the time of testing (Table 1). The participants did not use any special programs like beamforming or frequency compression in their hearing aid at the time of testing.

**Table 1.** Demographic Details of the Participants and Hearing Devices.

No.	Age (yr)	CI experience (yrs)	CI processor	CI lowest frequency boundary (Hz)	Contralateral HA	HA experience (yrs)
*1*	*53*	*2.6*	*Cochlear, CP910*	*563*	*Oticon Vigo Pro*	*28*
*2*	*59*	*1.11*	*Advanced Bionics* *Q 90*	*250*	*Phonak Naida, vented 0.6–0.8 mm*	*26*
*3*	*42*	*7*	*Medel Sonnet*	*750*	*Phonak Naida*	*10*
*4*	*73*	*3*	*Advanced bionics, Q90*	*250*	*Phonak Naida, vented 0.6–0.8 mm*	*18*
*5*	*65*	*10*	*Medel Duet 2*	*639*	*Phonak*	*>2*
*6*	*63*	*1.2*	*Cochlear CP910*	*313*	*GN resound*	*>2*
*7*	*75*	*0.6*	*Cochlear CP1000*	*438*	*Widex*	*>2*

**Figure 2.**
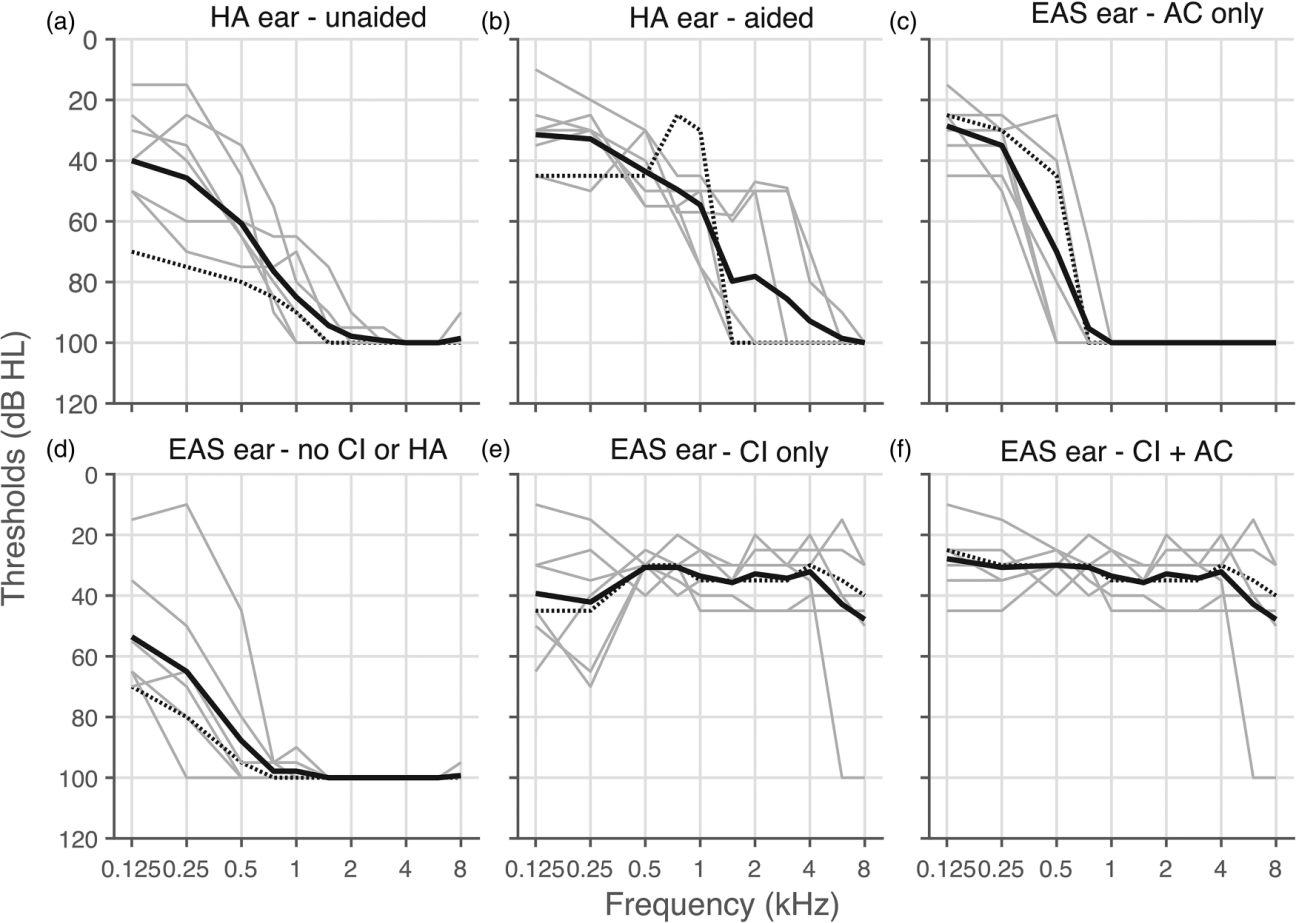
**Hearing thresholds.** (a), (d) Unaided thresholds for (a) the hearing-aid ear and (d) the EAS ear. (b), (c), (e), and (f) Aided thresholds for (b) the hearing-aid ear, (c) the acoustic stimulation of the EAS ear, (e) the electrical stimulation of the EAS ear, and (f) combined electrical and acoustical stimulation. Gray solid lines indicate thresholds for individual participants, black lines indicate the mean across participants, dashed solid black lines indicate the thresholds for participant P6 discussed as an example in the text. HA – hearing aid; EAS – electro-acoustic stimulation; AC – the acoustic part of the EAS device is turned on; CI – the electric part of the EAS device is turned on. Thresholds worse than 100 dB were assigned a value of 100 dB.

One of the participants (P1) had limited peripheral vision caused by Usher type 2A syndrome, while the other participants had normal, or corrected to normal, vision. Hearing thresholds (Figure 2) were measured up to 100 dB HL (a value of 100 dB HL was assigned if thresholds were worse). While testing one ear, the other ear canal was plugged (3M Ear, Taper Fit Earplugs, Maplewood, USA) with an attenuation of 32 dB and the ear was muffed with Optime 3 Peltor (3M, Maplewood, USA) that has an attenuation of 35 dB, which produced 40 dB of effective attenuation. Both the unaided hearing-aid side (Figure 2a) and unaided EAS-side thresholds (Figure 2d) were determined for pure tones through headphones. The aided thresholds (Figure 2b, c, e, f) were determined for warble tones as these tones do not produce standing wave patterns in the room. Note that thresholds to warble tones are similar to pure-tone thresholds for the frequencies of interest in our study (within 2 dB; Dockum & Robinson, 1975; Lentz et al., 2017).

Hearing devices improved low frequency audibility (<500 Hz) on average by 13 dB in the hearing-aid ear (compare Figure 2a & b) and by 40 dB in the EAS ear (cf. Figure 2d & f) and high frequency audibility (>500 Hz) in the EAS ear by 64 dB (cf. Figure 2d & f). Note that the implant also provided audibility to low frequencies (mean thresholds at 37 dB HL for frequencies of 125, 250, and 500 Hz, compare Figure 2d and e) where the acoustic part of the EAS also operated (Figure 2c). It indicates that for most of our participants there was a maximum overlap between the electric and acoustic stimulation in the EAS ear as permitted by the device. The thresholds with the EAS device (Figure 2f) were obtained by taking the best of the thresholds of the electric- (Figure 2e) and acoustic- (Figure 2c) only conditions.

The hearing aid and the cochlear implant were fitted according to local clinical guidelines. All participants included in the study are clinically monitored at RadboudUMC clinic and reported their device settings to be satisfactory at the start of the measurements. Experiments were done with their daily device settings without any refitting.

Some of the data from one participant (P5), who had the best low-frequency residual hearing in both ears in this participant group, have been published separately as a case study (Sharma et al., 2019).

### Set-up

Sound-localization experiments were carried out in a dark sound-treated room with a background noise level below 30 dBA (Van Bentum et al., 2017). The room consisted of a spherical wire structure with 115 speakers, positioned on an orthogonal double-pole azimuth-elevation spherical grid (Knudsen et al., 1979) with 5-deg spacing between the speakers along the cardinal axes, and 15-deg spacing for the other speaker locations. Sounds were presented in random order through randomized speaker locations, selected via custom-made MATLAB (Mathworks Inc.) scripts. Sound presentation and data acquisition were done through TDT system 3 hardware (Tucker Davis Technologies, Alachua, FL, USA), operated with custom-written MATLAB code. Head movements were recorded using a magnetic search coil technique (Robinson, 1963). The room had three perpendicular pairs of coils along its edges that generated high-frequency oscillating magnetic fields that were used to record the orientation of the search-coil. A green LED was mounted at the center of each speaker that was used for the coil-calibration procedure (next section), prior to the sound-localization experiment.

#### Calibration of Head Movement Tracking Coil

Participants sat in the center of the setup and wore a light-weight spectacle frame equipped with a head-fixed laser pointer. This pointer ensured that participants kept their gaze centered during the pointing tasks. The search coil was mounted on the nose bridge of spectacle frame and the participants visually pointed the laser to each of 24 known LED positions for the coil-calibration procedure. These responses were used to train two three-layer back-propagation neural networks that transformed the head-movement data (in mV) into calibrated azimuth and elevation angles (in deg, Bremen et al., 2007; Van Barneveld & Van Wanrooij, 2013).

### Stimulus

Sound stimuli were derived from 150-ms long white-noise bursts with 5-ms sine-squared onset and cosine-squared offset ramps. Sounds were bandpass-filtered in one of four ways: 100–400 Hz (low frequency, LF), 500–1500 Hz (mid-frequency, MF), 3000–20000 Hz (high frequency, HF), and 500–20000 Hz (mid-to-high frequency, MHF). The four sound stimuli were interleaved in one block and presented at a level of 65 dB(A) from one of 16 locations with four repetitions per speaker location with a total of 64 trials per listening condition. Five listening conditions were tested and a description of each condition is presented in the section “Listening Conditions.” Speaker locations were randomly selected between −80 deg and +80 deg in azimuth and between −45 and +60 deg in elevation. Participants took part in a familiarization task of 20 trials before the start of the actual experiment.

### Paradigms

A trial began with the presentation of a green fixation LED from the straight-ahead location, at [0,0] deg. The participant had to point the head-fixed laser toward this LED. This ensured that the starting position was the same for each trial. The participant was provided with a small hand-held button box to start each presentation. Pressing the button turned off the fixation light within 100 to 300 ms and started the sound stimulus 200 ms after that. Participants were instructed to orient their head as quickly and as accurately as possible and direct the head-fixed pointer in the perceived direction of the stimulus. Because reaction times exceeded 150 ms (the duration of the stimulus) and no visual feedback was provided, all responses were made under complete open-loop conditions.

### Listening Conditions

To test the effects of the devices and residual hearing capabilities on sound localization, we tested five listening conditions:

- A-EX. Bimodal stimulation with acoustic stimulation at the non-implanted side (A), electric (E) stimulation and an open ear canal (X) at the implant side (seven participants).
- A-EA. Bimodal EAS stimulation with acoustic amplification in the non-implanted ear and electro-acoustic stimulation in the implanted ear (seven participants).
- X-EX. Electric stimulation only without acoustic amplification; open ear canal at both sides (five participants). The implant was not re-fitted after taking the acoustic component of the EAS device out of the ear canal.
- X-EA. Electro-acoustic stimulation only with the hearing aid at the non-implanted side taken out, leaving the ear canal open (six participants).
- A-XA. Bilateral acoustic amplification with electric stimulation turned off. All seven participants performed the localization task with this listening condition.

We chose to measure with open ear canals (X) to have a large range of hearing asymmetry across participants due to the natural variation in residual hearing (Figure 2). Occluding the ear canals would have yielded purely monaural listening conditions limiting the variation in hearing asymmetry.

Each listening condition was presented in a separate experimental block. The order of the blocks was randomized across the participants. Some of the participants did not complete all blocks due to time limitations.

### Analysis

A custom-written MATLAB program was used to automatically detect head saccades with velocities exceeding 20 deg/s. Visual inspection allowed for manual adjustment of the end position of the saccades. These end positions (in deg) were taken as the participant's response, where positive azimuth angles indicate positions to the implanted ear while negative azimuth angles indicate responses to the hearing-aid ear.

As most responses were toward either the EAS or the hearing-aid ear without a clear linear or continuous stimulus-response relationship (see Results), we quantified the proportion of responses toward the implanted ear, *p_EAS_*, as a measure of localization bias (note that “EAS” in *p_EAS_* is used to indicate the side of the implanted ear, even for listening conditions without electrical and/or acoustic stimulation). This continuous variable can vary from 0, indicating all responses were directed to the hearing-aid ear, to 1, indicating that all responses were directed to the implanted side. A *p_EAS_* value of 0.5 indicates there is no localization bias, with 50% of the responses being directed toward the implanted side, and the other half to the contralateral side.

We also calculated the more-commonly used root-mean-squared localization error (*E_RMS_*, in deg) for comparison to other studies. For normal-hearing listeners, this error is typically less than 10 deg (Dorman et al., 2014). Chance level error depends on the listener's response strategy. If a listener is unable to perceive sound location and then localizes randomly within the stimulus range, the error will be 73 deg. If that listener does not respond and keeps on looking straight ahead, then the error will be 46 deg.

Participants were unable to localize sound elevation accurately (data not shown). Sounds were localized around 0 deg in elevation (i.e., on the horizontal plane), irrespective of target location, sound frequency, listening condition or participant. This is expected from listeners that do not have access to spectral pinna cues and we do not discuss this further.

#### Hearing Asymmetry

**Figure 3.**
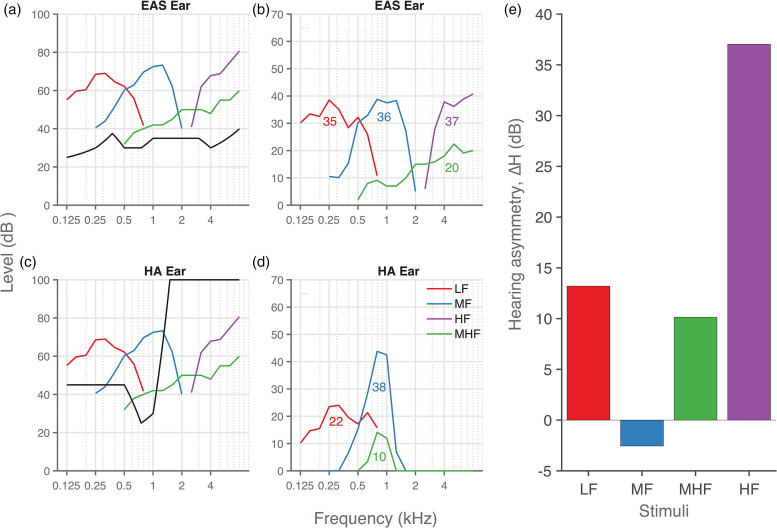
Hearing asymmetry. (a), (c) Aided hearing thresholds (black lines) and sound level (colored lines) of the four stimuli in dB HL for the (a) EAS and (c) hearing-aid ear. (b), (d) Effective audibility of each noise band in the (b) EAS ear and (d) hearing-aid ear, with the averaged audibility across frequencies (shown as inset numbers). (e) Hearing asymmetry (Δ*H*) was calculated by subtracting the audibility in the hearing-aid ear from that in the EAS ear. Data from participant P6.

Hearing asymmetry (Δ*H*) between ears was obtained by calculating the audibility of the four sound stimuli for each of the five listening conditions (exemplified by data from participant P6, Figure 3).

First, the sound levels of the stimuli were converted from dB SPL to dB HL in the following way. We measured the dB SPL sound level in 1/3^rd^ octave bands using a sound level meter (Brüel & Kjær, Denmark, 2236) with a free-field microphone (type-4191) positioned at the location of the participant's head. Then, correction was applied in gammatone filter banks (Slaney, 1993) to compensate for the bandwidth of the noise burst. Finally, the corrected dB SPL sound level was converted to dB HL (Figure 3a and c, colored curves) using standard correction factors (Champlin & Letowski, 2014).

Second, hearing thresholds in dB HL for both ears (black curves in Figure 3a and c) were then subtracted from the noise-burst sound level to determine audibility of each noise burst per frequency (Figure 3b and d). Negative differences, indicating no audibility, were set to zero. Then, the acoustic power was summed across frequencies for each of the four noise bursts to obtain a measure of the overall audibility of each stimulus type (Figure 3b and d; inset numbers).

Finally, the hearing asymmetry (Δ*H*) was obtained by subtracting the audibility in the hearing-aid ear from the audibility in the implanted ear (Figure 3e). A positive hearing asymmetry thus indicates better audibility in the implanted ear. Quantification of the hearing asymmetry for all the other participants is shown in Supplemental Material (Figures. S1-1 to S1-7). In general, hearing asymmetry favored the implanted ear, which is likely due to the audibility over a broader range of frequencies in the implanted ear (< 8 kHz) in comparison to the hearing-aid ear (< 2 kHz).

#### Logistic Regression

We performed logistic regression to fit the localization bias, *p_EAS_*, as a function of hearing asymmetry (Δ*H*)**:**

(1) $$p_{EAS}=\;{\left(1+e^{\frac{-2ln9}{\omega }\Delta H}\right)}^{-1}$$

with width, *ω* (dB), the hearing-asymmetry range for which *p_EAS_* varied from 0.1 to 0.9. This is the range of hearing asymmetry that should yield extreme bias on either side with *p_EAS_* of 0.1 and 0.9 indicating a strong bias toward the hearing-aid and implanted ear respectively.

Large values of *ω* indicate that hearing asymmetry barely influences sound localization. This equation implies mirror symmetry of localization bias, when hearing is symmetric (i.e., *p_EAS_* = 0.5 for Δ*H *= 0). The data, however, indicated that sound localization was biased towards the hearing-aid ear contralateral to the implant ear (*p_EAS_*<0.5), even when audibility was symmetric (Δ*H* = 0). Therefore, we adjusted this model for listening conditions with the hearing aid turned on by including a hearing-aid preference, *θ* (in dB):

(2) $$p_{EAS}=\;{\left(1+e^{\frac{-2ln9}{\omega }(\Delta H-\theta )}\right)}^{-1}$$

The hearing-aid preference measures the hearing asymmetry for which *p_EAS_* is 0.5, when the hearing aid is turned on. Positive or negative values of *θ* indicate that sound localization is biased toward the hearing-aid or EAS ear, respectively, more than expected by hearing asymmetry, ΔH, alone – i.e., responses to either the hearing-aid, or the EAS ear, are favored.

The fitting (for general principles and parametrization, see Kuss et al., 2005; for details on the sampling parameters and sampling checks used, see van de Rijt et al., 2019) was achieved by Bayesian inference using a Markov Chain Monte Carlo technique implemented in JAGS (Plummer, 2003) through MatJags (Turner et al., 2013). Posterior distributions of parameters *ω* and *θ* were obtained for each participant separately, but also hierarchical group-level parameters were obtained. Equations 1 and 2, and individual and group-level parameters were all fitted simultaneously.

#### Parameter Estimation

We determined the mean and 95% confidence interval (95%-CI) for the error (*E_RMS_*), the bias (*p_EAS_*) and hearing asymmetry (ΔH). The 95%-CIs were obtained through bootstrapping using 1000 samples.

From the distribution of the fitted parameters (Equations 1 and 2) we determined the mean and the 95% highest-density interval (95%-HDI) of the width and preference (hearing-aid bias). We discuss these estimates to assess how hearing asymmetry influences sound localization and how much the hearing-aid ear is preferred over the EAS ear.

The effect sizes and 95%-CI or 95%-HDI are reported as effect size [95%-CI/HDI lower bound, upper bound].

## Results

### Sound Localization Behavior of a Typical Bimodal EAS User

**Figure 4.**
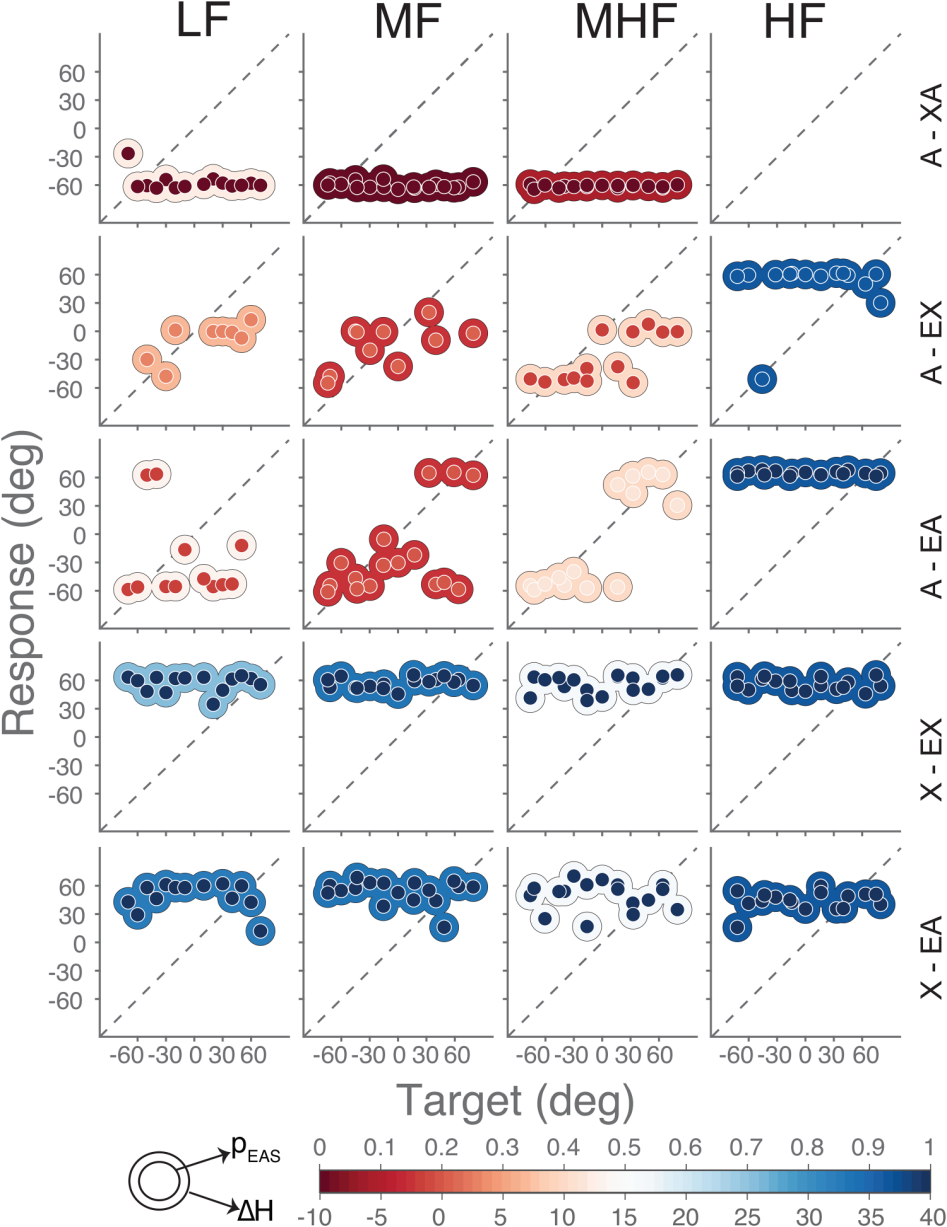
Sound localization of participant P6. Target-response plots are shown for the five listening conditions (rows) and four sound types (columns). Rows correspond to bilateral acoustic only (A – XA), bimodal (A – EX), best aided (A – EA), cochlear implant only (X – EX), and electro-acoustic only (X – EA). Columns correspond to low-frequency (LF), mid-frequency (MF), mid-to-high frequency (MHF) and high-frequency (HF) sounds. Note that no HF sounds were heard by this participant in the bilateral-acoustic A-XA listening condition (top, right). Colored circles indicate single responses. Colors of inner circles represent the magnitude of localization bias (*p_EAS_*) while colors of outer circles represent the magnitude of hearing asymmetry (Δ*H*). Note that the center (0) for *p_EAS_* and hearing asymmetry Δ*H* are color-coded differently for visualization purposes only. Positive Δ*H* indicates better audibility in the implanted ear.

Bimodal EAS users had to localize sounds consisting of various frequency bands in different listening conditions in which their devices were independently turned on or off. These two experimental manipulations (frequency bands and listening condition) systematically affected a participant's hearing asymmetry. The goal of this study was to see whether this also affected sound localization. We will first show that this was indeed the case for one of our participants (P6) with whom we measured all conditions (Figure 4). For the majority of listening conditions and sounds, P6 responded to only one side. When the implant was turned off (Figure 4, top row), sounds were perceived consistently on the hearing-aid side (localization bias, *p_EAS_* = 0, red inner circle) for all sounds. When the hearing aid was turned off (bottom two rows), sounds were judged exclusively to the implanted side (*p_EAS_* = 1, blue inner circle). Both findings are in line with the hearing asymmetry induced by turning either device off (blue outer circle for hearing aid off with ΔH from + 16 to + 37 dB, red outer circle for implant off with ΔH from −27 to + 13 dB). However, hearing asymmetry typically favored the implant (the average ΔH across all listening conditions was 16 dB), which was not reflected by the localization bias (*p_EAS_*  = 0.6).

High-frequency sounds (Figure 4, fourth column) were, if heard, almost always localized toward the implanted side (*p_EAS_* = 1, blue inner circle; except for one response). As these sounds were not audible by the (unaided or aided) ear on the hearing-aid side (Figure 3b and d, purple curve), the hearing asymmetry was Δ*H* =  + 37 dB (Figure 4, blue outer circle). As these sounds were also inaudible through the acoustic part of the EAS device, participant P6 reported not hearing any sound while listening with both acoustic devices alone (A-XA).

While listening bimodally (A-EX) or with bimodal EAS (A-EA), the participant had a localization bias toward the hearing-aid ear (*p_EAS_* between 0.15 and 0.43) for the three other sounds (LF, MF, and MHF), but did not localize all sounds toward either extreme. For these sounds and these listening conditions, hearing asymmetry was not as extreme as for the high-frequency sounds; also, intermediate levels of hearing asymmetry were observed (Δ*H* between −2 and 13 dB; Figure 4, from red to white outer circles). The hearing asymmetry and localization bias were correlated with each other for these mid-frequency and mid-to-high frequency sounds in the bimodal EAS and bimodal conditions (Figure 4, middle row, A-EA for MF and MHF; note the matching inside–outside colors).

Overall, the participant did not localize sounds accurately in any of the listening conditions (as illustrated in Figure 1c and d). Localization errors tended to be large (*E*_RMS_ > 30 deg), even when listening with all devices turned on (A-EA). Nevertheless, listening condition and noise-burst frequency systematically affected both hearing asymmetry and sound-localization bias of this participant. This happened in a similar, but not identical, way for both measures (inner and outer colors match to a large extent). Hearing asymmetry generally favored the implant more than the localization bias (which is why the color scales in Figure 4 included an offset for Δ*H*, in order to align the color schemes).

In general, the other participants (Supplemental Figures. S2-1 to S2-7) localized sounds in a similar fashion with incorrect responses either to the side with hearing aid or to the side with cochlear-implant depending sound frequency and listening condition. Only the mid and mid-to-high frequency sounds in the bimodal EAS and bimodal listening conditions were localizable to some extent, with considerable response bias and variability.

### Localization Error

**Figure 5.**
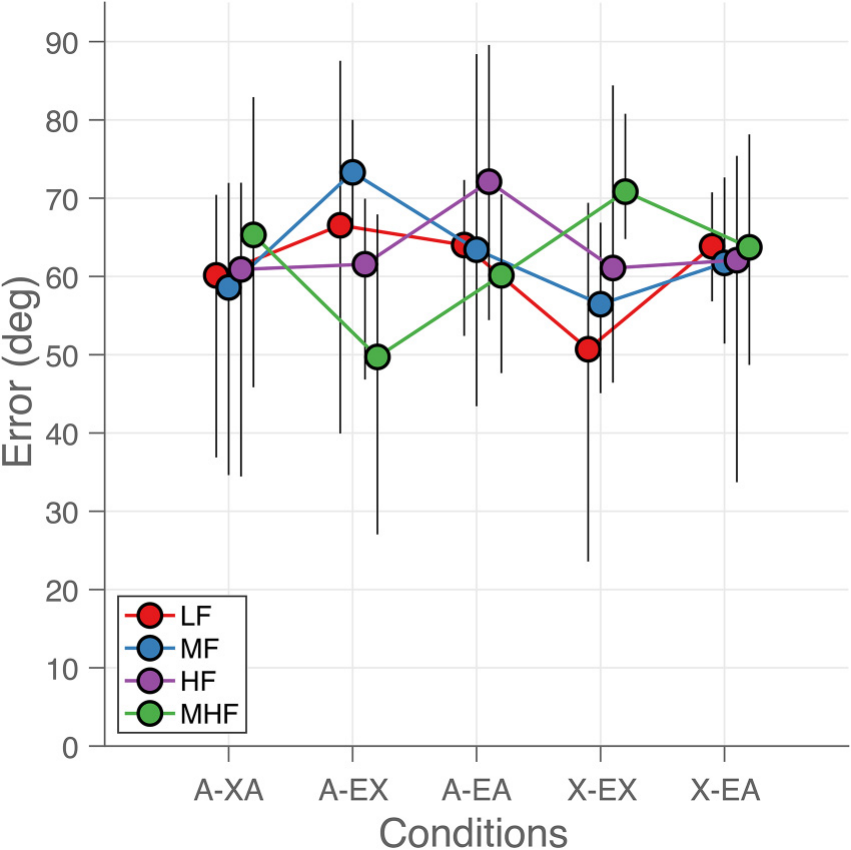
Sound localization errors. Average root-mean-square localization error is plotted as a function of listening condition (abscissa) and for each sound type (indicated by color and connected by lines). Each colored dot represents the mean across the participants in each listening condition and for each sound type. The error bars indicate the 95% confidence intervals around the mean. The curves for each sound type are slightly shifted for visual purposes.

Overall sound localization performance was inaccurate (Figure 5) for all listening conditions and frequency bands. The localization errors were large and did not depend on listening condition or frequency band (Figure 5). The average *E*_RMS_ for all listening conditions and sound types was 62 deg (95%-CI = [58, 66] deg). We observed no overall benefit of acoustic amplification by the EAS in the bimodal EAS condition (A-AE), when compared to the bimodal condition (A-XE). For example, the errors for mid-to-high frequency sounds (MHF) were comparable at 50 deg (95%-CI = [26, 68] deg) and 60 deg (95%-CI = [46, 70] deg), respectively. However, localization errors varied considerably across participants. For example, in the extreme case of participant P5, the localization errors for the low-frequency sounds (LF) were close to normal-hearing (*E*_RMS_ of 12 deg, more details for this participant can be found in Sharma et al., 2019).

### Hearing Asymmetry and Localization Bias

**Figure 6.**
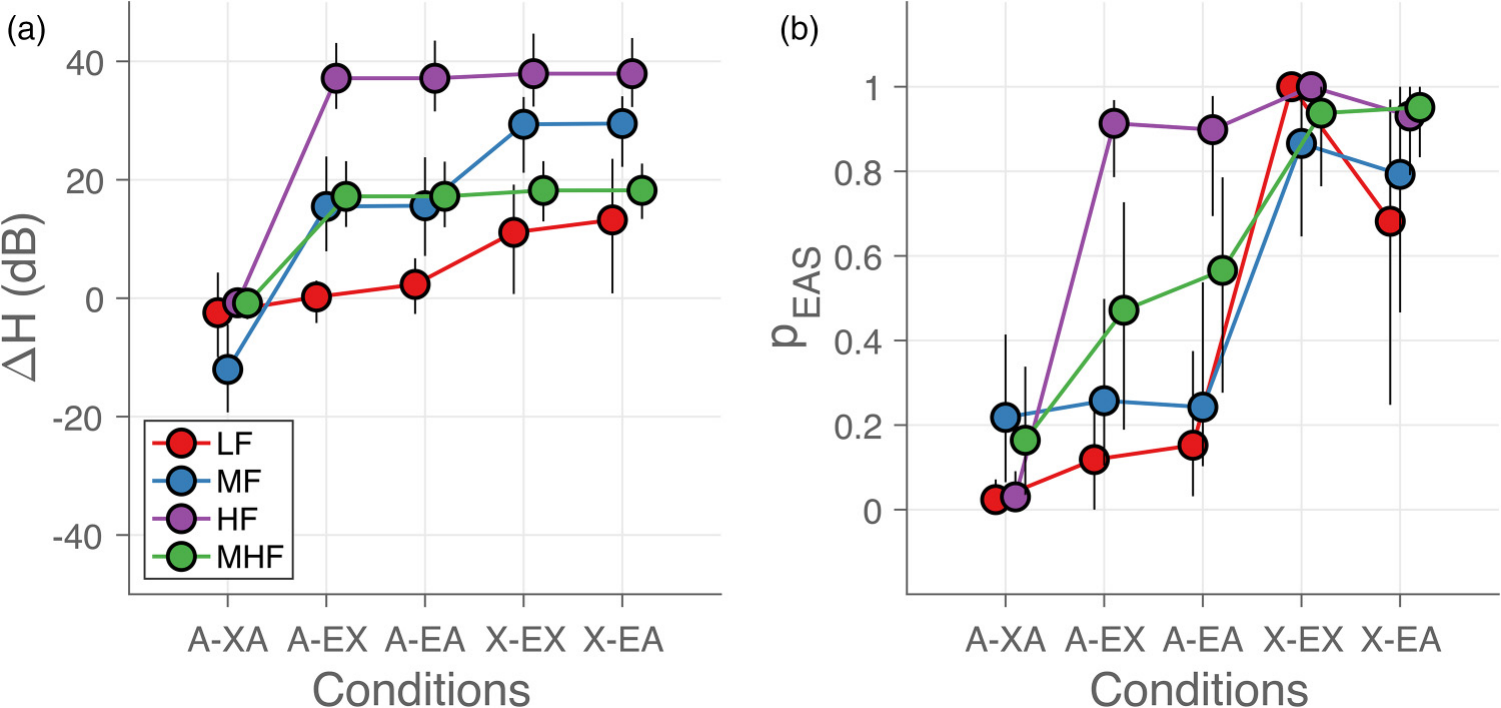
Hearing asymmetry and localization bias. (a) Hearing asymmetry (Δ*H*) and (b) mean probability of responding toward implanted ear (*pEAS*) are shown as a function of listening condition. Each colored dot represents the mean across the participants in each listening condition and for each sound type. The error bars indicate the 95% confidence interval across participants. The curves for each sound type are slightly shifted for visual purposes.

In contrast to the lack of systematic changes in localization errors, hearing asymmetry (Figure 6a) and localization bias (Figure 6b) were systematically affected both by listening condition and sound-frequency bandwidth. Hearing asymmetry (see Methods, Figure 3) systematically increased as the bandwidth of the sounds changed from low (LF, red) to high (HF, purple). Hearing asymmetry also changed with listening condition, with asymmetry favoring the hearing-aid side for acoustic-only listening (Figure 6a, A-XA), and the EAS side for EAS-only listening (Figure 6a, X-EA).

Localization of low- and mid-frequency sounds (LF - red and MF - blue) was on average biased toward the hearing-aid side (*p_EAS_*< 0.0 – 0.4) when the contralateral hearing aid was turned on (A-XA, A-EX, and A-EA). When turned off (X-EX and X-EA), however, the bias switched toward the EAS side (*p_EAS_* > 0.5). Response bias for mid-to-high-frequency noise bursts (MHF, green) shifted from the hearing-aid ear to the EAS ear as the listening condition changed from the bilateral-acoustic (A – XA) to the EAS-only (X – EA) listening condition, with little average bias (*p_EAS_* around 0.5) for the bimodal (A – EX) and bimodal EAS (A – EA) conditions. The high-frequency sounds (HF, purple) were always perceived on the EAS side except when the implant was turned off (A – XA).

Overall, we obtained a systematic interaction of stimulus type and listening condition on the sound localization bias. Localization of low-frequency sounds was typically dominated by the contralateral hearing aid (if turned on), while high-frequency sounds were largely localized toward the EAS side (if turned on). Mid-to-high-frequency sounds were more likely perceived with a smaller bias, especially for the bimodal EAS and bimodal hearing conditions. This finding largely corresponds to the changes in hearing asymmetry (cf. Figure 6a and b).

### Localization Bias Depends on Hearing Asymmetry

**Figure 7.**
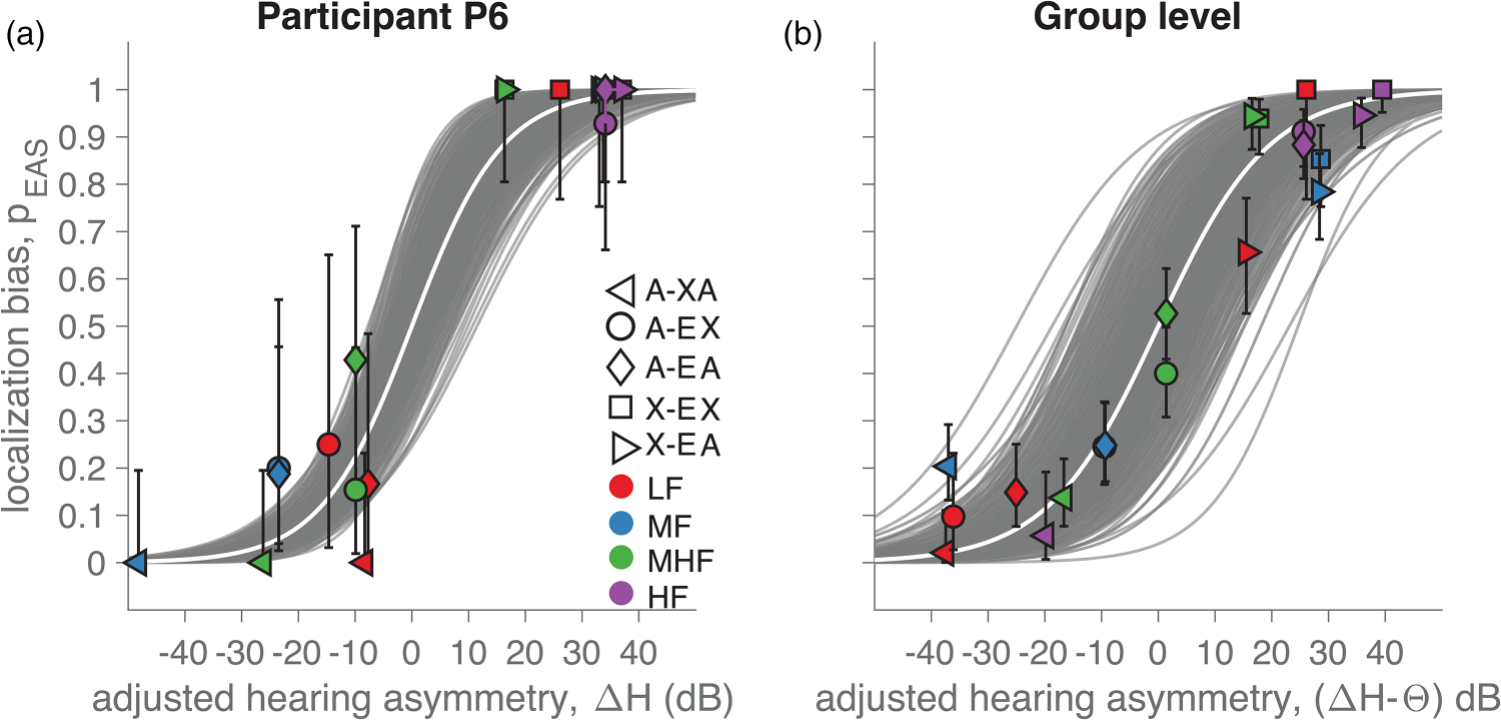
Localization bias as a function of hearing asymmetry. (a) Example from participant P6. (b) Grand average across participants. The noise-burst bandwidth is indicated with colors, and listening conditions with marker types. Error-bars show the binomial 95% confidence interval. The gray curves are credible logistic fits using Bayesian estimation on Equation 2, with the white curve indicating the best fit. Note that the hearing asymmetry, Δ*H*, was adjusted by subtracting the most-likely value of *θ*, obtained from Equation 2. As such, the hearing-aid preference is already taken into account, and the curves are centered at [Δ*H*, *p_EAS_*] = [0 dB, 0.5]. Positive Δ*H* indicates better audibility in the implanted ear.

Hearing asymmetry is a continuous variable, incorporating experimental factors like listening condition and frequency band and the continuous data descriptors like hearing thresholds. As such, it may be a more informative variable compared to the unaided hearing thresholds alone to explain the underlying differences in observed localization bias resulting from listening condition, sound type and hearing thresholds. We tested this assumption by plotting localization bias as a function of hearing asymmetry and fitting the logistic curve of Equation. (2). Figure 7a shows the result of this analysis on the data for participant P6, while Figure 7b shows the data for all participants. The average width of the curve (ω; Figure 7b), which represents the amount of hearing asymmetry needed to vary the localization bias from 0.1 to 0.9, was 43.4 dB (95%-HDI = [33.4, 52.1] dB).

Thus, hearing asymmetry systematically affected the localization bias, both for participant P6 (Figure 7a) and at the group level (Figure 7b). Sounds that are more audible in the EAS ear were more likely judged on the EAS side and toward the hearing-aid side if audibility was superior in that ear. Note that the model of Equation (2) includes the hearing-aid preference, *θ*. In Figure 7b, we adjusted the hearing asymmetry with the average estimate of *θ* for each participant for visual purposes. The hearing-aid preference was quite substantial (mean *θ* = 18.8 dB, 95%-HDI = [11.3, 26.1 dB]). This suggests that bimodal EAS users have a large preference to localize sounds toward the hearing-aid ear, even when hearing is symmetric.

## Discussion

### Summary

We investigated how bimodal EAS users localize sounds of varying frequency content with or without their hearing devices turned on. Overall, our bimodal EAS users localized sounds poorly (Figure 5). When the bimodal listeners were using both devices, their responses were biased toward the implanted side for high-frequency sounds and to the hearing-aid side for low-frequency sounds (Figure 6b, A-EA). Similarly, turning off either the acoustic or the electrical part of the EAS biased the responses toward the hearing-aid side (Figure 6b, A-XA and A-EX) and turning off the hearing-aid introduced a response bias toward the EAS side (Figure 6b, X-EX and X-EA). For mid- and mid-to-high-frequency sounds, the response bias varied considerably between listeners (Figure 6b, blue and green, note the large error bars). The individual frequency-specific hearing asymmetry (Figures. 6a) was a good predictor for these biases and their variability (Figure 7), in line with our predictions made in the rationale (Figure 1). However, an inherent preference to localize sounds toward the hearing-aid side was observed that could not be predicted solely from hearing asymmetry. In the next sections, we will contextualize these findings in relationship to earlier studies investigating sound localization by bimodal EAS users, expand on the availability and use of sound-localization cues by these users, discuss how hearing asymmetry plays a role, and discuss the origin of the hearing-aid preference.

### Bimodal EAS Sound Localization

On average, the bimodal EAS users in our study localized sounds poorly (*E_rms_* of 62 deg), especially when compared to previous studies (25, 45 and 13 deg in Dunn et al., 2010; Gifford Dorman et al., 2014; Kortje et al., 2020, respectively). Several reasons could underlie this difference in performance. First, difference in experimental setups and specifically, the range of speakers may trivially affect the error measure. For example, in Dunn et al., 2010, speaker locations ranged from −54 to + 54 deg, while ours ranged from −80 to + 80 deg. Thus, for the former the *E_rms_* of chance responses could range from 32 deg to 63 deg, while for our study it ranges between 48 deg and 73 deg. Second, top-down cognitive factors and strategies (e.g., influenced by instruction, task, speaker layout and (in)visibility) may introduce significant experimental differences (Arras et al., 2022). Third, sounds of different frequency content, duration, and power spectra have been used (speech vs broadband white noise vs narrowband sounds). Fourth, all studies employed participants with large differences in hearing preservation and these might explain performance variability across studies. For example, hearing preservation was better in Dunn et al., 2010 and Kortje et al., 2020 than for our participants, implying better hearing thresholds and a larger hearing symmetry, which could lead to lower errors. Note that the participants in Dunn et al., 2010 were implanted with shorter 10 mm electrodes than for our participants and this might account for larger hearing preservation in their participant group. Overall, all the above factors could explain the discrepancies in outcome, but the frequency information of the stimuli, amount of hearing preservation and hearing asymmetry should affect sound-localization performance specifically according to our rationale (Figure 1).

### Low-Frequency Sound Localization

We hypothesized that bimodal EAS users can potentially localize low-frequency sounds accurately (Figure 1). These bimodal users typically have good and symmetric (aided) hearing thresholds, which should allow for accurate processing of ITDs and low-frequency sound localization. Indeed, earlier studies have demonstrated that many bimodal EAS users are sensitive to ITDs (Gifford Grantham et al., 2014; Gifford & Stecker 2020; Kortje et al., 2020) and are able to localize sounds in the horizontal plane by relying on ITDs (Gifford Grantham et al., 2014; Kortje et al., 2020). Despite these previous findings and low-frequency hearing preservation (Figure 2), the listeners in this study typically experienced great difficulties in localizing low-frequency sounds (in line with the general poor performance as discussed in the previous section). Only one participant could do this successfully for low-frequency (100 − 400 Hz) sounds when using both devices (P5; Sharma et al., 2019; Supplemental Figure S2-5, A-EA, LF), while two more could also localize mid-frequency (500−1500 Hz) sounds to a reasonable extent (P1, P3, and P5; Supplemental Figures. S2-1,3,5, A-EA, MF).

The insensitivity to ITDs for many bimodal EAS users (this study, but see also examples in Dunn et al., 2010; Gifford Grantham et al., 2014; Kortje et al., 2020) may be caused by a combination of factors other than poor low-frequency hearing preservation and hearing asymmetry. These perturbing factors may include (i) effects of hearing impairment not restored by the devices (e.g., temporal smearing, broadening of temporal auditory filters), (ii) differences in processing delays across the devices preventing binaural integration (Dorman et al., 2016; Francart et al., 2009; Hassager et al., 2017; Lenssen et al., 2011; Zirn et al., 2018, 2019), (iii) the presence of a stiff structure, i.e., the electrode, perturbing cochlear mechanics, and iv) a large spread of electrical excitation by the cochlear implant causing spectral smearing (Fu & Nogaki, 2005; Tang et al., 2011). Overall, given these fundamental challenges, it is remarkable that some bimodal EAS listeners are sensitive to ITDs and can localize low-frequency sounds (Dunn et al., 2010; Gifford Grantham et al., 2014; Gifford & Stecker 2020; Kortje et al., 2020).

Another factor may be that these bimodal EAS users weight ITD cues less than ILD cues when forming a spatial percept. Evidence that supports this notion comes from turning off the electrical and acoustic parts of the EAS device. Fine structure ITDs are only provided by the acoustic hearing devices and not by the cochlear implant. Therefore, turning off the electrical part should not influence sound localization if the listener fully relies on ITD. However, turning off the implant did influence sound localization for the three “good” performers (Supplemental Figures. S2-1,3,5; compare A-EA with A-XA for LF and MF sounds). Furthermore, disabling the acoustic part of the EAS device did not affect sound localization to a large extent (Supplemental Figures. S2-1,3,5; compare A-EA with A-EX for LF and MF sounds) indicating that our participants did not use fine-structure ITDs.

These findings are in line with the idea that low-frequency ILDs and ITDs are both weighted in creating a spatial percept. Other studies have observed (mainly in normal-hearing listeners) that (re)weighting of cross-frequency sound localization cues can be acute and adaptive (Van Wanrooij & Van Opstal, 2004, 2005, 2007; Dahmen et al., 2010; Dahmen et al., 2010; Goupell & Stakhovskaya, 2017, 2018; Klingel & Laback, 2022). Questions such as whether this plays an important role for hearing-impaired individuals using different types of hearing devices (such as bimodal EAS users) and whether such weighting reflects optimal use of sub-optimal cues warrant further investigation.

### Hearing Asymmetry Biases Sound Localization

Sound localization was strongly biased for most listening conditions and frequency bands of the noise bursts (Figure 6b). For the bimodal EAS condition, high-frequency sounds that were audible only in the EAS ear (Figures. 1a, right, 2, 6A) were localized towards that side (Figure 6b, A-EA, purple); low-frequency sounds that were almost equally audible in both ears (Figures. 1a, left, 2, 6A) tended to be localized toward the hearing-aid side (Figure 6b, A-EA, red); and mid-and mid-to-high-frequency sounds that were better audible in the EAS ear (Figures. 1a, center, 2, 6A) tended to have little bias, albeit with large cross-individual variability. These biases were related to the hearing asymmetry of each listener in the bimodal EAS condition (Figure 7b), as quantified from differences in the individual hearing thresholds (Figure 2) of each ear for each frequency band and listening condition. This relationship is mostly in line with the predictions explained in the Introduction (Figure 1). Yet, there are some notable exceptions. First, our listeners preferred to respond toward the hearing-aid side, which will be discussed in the following section. Second, low-frequency sounds may be localized quite accurately by some listeners (as discussed in the previous section and as reported in Loiselle et al., 2015). Finally, hearing asymmetry led to a consistent bias, which was not reduced even with long-term exposure to this hearing asymmetry. This is in stark contrast to our expectation that bimodal EAS users could potentially use perturbed low-frequency ILDs in the absence of ITDs (i.e., for mid-frequency sounds; Veugen Chalupper et al., 2016; Loiselle et al., 2015; Gifford & Stercker 2020; Sharma et al., 2021). Evidence from other studies suggests that a bias induced by acute asymmetric hearing may be overcome by long-term exposure or adaptation (Van Wanrooij & Van Opstal, 2007; Kumpik et al., 2010, in acutely plugged normal-hearing: Agterberg et al., 2018; Agterberg, Hol, et al., 2011; Agterberg, Snik, et al., 2011, in participants with bone-conduction devices; Van Wanrooij & Van Opstal, 2004, participants with single-sided deafness). One explanation of why bimodal EAS users cannot reduce this localization bias may be that the hearing asymmetry changes for every sound. This inconsistency may impede adaptation.

### Preference for Hearing Aid

We observed a preference for listeners to localize sounds toward the non-implanted ear using the hearing aid. The observed hearing asymmetry had to be adjusted by about 19 dB in favor of the hearing-aid ear (equation (2); Figure 7). This preference was not observed in earlier studies (Dunn et al., 2010; Veugen et al., 2016, 2017; Kortje et al., 2020; Sharma et al., 2021; Gifford Dorman et al., 2014). Whether the preference was really absent, is hard to determine as the localization bias itself depends on individual hearing abilities and on the frequency content of the target sounds.

The observed preference toward the hearing-aid side is not predicted by our hearing-asymmetry model (Figure 1). It might be due to a subjective preference because of stimuli ‘sounding better’ in the hearing-aid ear. Alternatively, objective factors—not included in our hearing-asymmetry measure—may influence binaural differences. For example, differences in loudness recruitment in both ears may cause a loudness imbalance (Francart et al., 2008; Oetting et al., 2016; Spirrov et al., 2018; Van Eeckhoutte, Spirrov, et al., 2018; Van Eeckhoutte Wouters et al., 2018; Veugen Chalupper et al., 2016). Similarly, a shorter processing time in the hearing aid may favor the hearing-aid ear (Francart et al., 2008; Francart & McDermott, 2013) and might be hard to overcome (Javer & Schwarz, 1995). Another possible explanation for the hearing-aid preference may be abnormal dichotic fusion across different frequencies, which has been shown to lead to lateralization even when there are no ITDs or ILDs (Reiss et al., 2014; 2017).

### Limitations and Challenges

Overall, our conceptual hearing-asymmetry model (Figure 1) could explain the localization behavior of bimodal EAS listeners, but several challenges remain. In general, the amount of residual hearing in our study was smaller than reported in other studies, which reduces the bimodal benefit. This may be due to the relatively strict criteria for implantation in the Netherlands. Only individuals with bilateral severe-to-profound hearing loss are eligible for implantation and they usually have limited functional hearing in the non-implanted ear. For e.g., one of our participants had very limited functional residual hearing in the implanted ear with unaided thresholds beyond 100 dB HL for 250 Hz and above, reducing the likelihood of EAS benefit.

For clinical purposes, a hearing instrument test with a coupler and real ear measurements immediately before the experiments could help to optimize fitting and potentially reduce hearing asymmetry. To further consolidate the correlation between hearing asymmetry and localization behavior, it would be useful to map the loudness growth function for both ears in response to the experimental stimuli. This would capture the fact that independently acting automatic gain control across the devices will produce different outputs according to the sound presentation level. For future experiments, it would also be interesting to use multiple sound levels as independently acting automatic gain control across the devices might interact with the hearing asymmetry as a function of presentation level.

## Conclusion

Hearing asymmetry in bimodal EAS users causes a frequency-dependent sound localization bias. Furthermore, our bimodal EAS users had a global preference to localize toward the hearing-aid side. Overall, our results imply that accurate localization of sounds with arbitrary spectra by bimodal EAS users with considerable frequency-dependent hearing asymmetry is hard to obtain.

## Supplemental Material

sj-docx-1-tia-10.1177_23312165221143907 - Supplemental material for Hearing Asymmetry Biases Spatial Hearing in Bimodal Cochlear-Implant Users Despite Bilateral Low-Frequency Hearing Preservation Click here for additional data file. Supplemental material, sj-docx-1-tia-10.1177_23312165221143907 for Hearing Asymmetry Biases Spatial Hearing in Bimodal Cochlear-Implant Users Despite Bilateral Low-Frequency Hearing Preservation by Snandan Sharma, Lucas H.M. Mens, Ad F.M. Snik and A. John van Opstal, Marc M. van Wanrooij in Trends in Hearing
