# Supplementary material for: Hearing Asymmetry Biases Spatial Hearing in Bimodal Cochlear-Implant Users Despite Bilateral Low-Frequency Hearing Preservation
Source: Trends Hear. 2023 Jan 5;27:23312165221143907. doi: 10.1177/23312165221143907 (PMC9829999; doi:10.1177/23312165221143907)
Supplement: sj-docx-1-tia-10.1177_23312165221143907 - Supplemental material for Hearing Asymmetry Biases Spatial Hearing in Bimodal Cochlear-Implant Users Despite Bilateral Low-Frequency Hearing Preservation [file sj-docx-1-tia-10.1177_23312165221143907.docx]

Supplementary material

# Hearing asymmetry

Hearing asymmetry (∆H, Fig. S1-1 to S1-7) between ears was obtained by calculating the audibility of the four sound stimuli for each of the five listening conditions (Fig. 3, data from participant P6 in main article).

Figure S1-1**. Computing hearing asymmetry**. (A,C) Aided hearing thresholds (black lines) and sound level (colored lines) of the four stimuli in dB HL for the (A) EAS and (C) hearing-aid ear. (B,D) Audibility of each noise band in the (B) EAS ear and (D) hearing-aid ear, with the averaged audibility across frequencies (inset numbers). (E) Hearing asymmetry (∆H) was calculated by subtracting the audibility in the hearing-aid ear from that in the EAS ear. Data from participant P6.

**Figure S1-2. Computing hearing asymmetry**. (A,C) Aided hearing thresholds (black lines) and sound level (colored lines) of the four stimuli in dB HL for the (A) EAS and (C) hearing-aid ear. (B,D) Audibility of each noise band in the (B) EAS ear and (D) hearing-aid ear, with the averaged audibility across frequencies (inset numbers). (E) Hearing asymmetry (∆H) was calculated by subtracting the audibility in the hearing-aid ear from that in the EAS ear. Data from participant P6.

**Figure S1-3. Computing hearing asymmetry**. (A,C) Aided hearing thresholds (black lines) and sound level (colored lines) of the four stimuli in dB HL for the (A) EAS and (C) hearing-aid ear. (B,D) Audibility of each noise band in the (B) EAS ear and (D) hearing-aid ear, with the averaged audibility across frequencies (inset numbers). (E) Hearing asymmetry (∆H) was calculated by subtracting the audibility in the hearing-aid ear from that in the EAS ear. Data from participant P6.

**Figure S1-4. Computing hearing asymmetry**. (A,C) Aided hearing thresholds (black lines) and sound level (colored lines) of the four stimuli in dB HL for the (A) EAS and (C) hearing-aid ear. (B,D) Audibility of each noise band in the (B) EAS ear and (D) hearing-aid ear, with the averaged audibility across frequencies (inset numbers). (E) Hearing asymmetry (∆H) was calculated by subtracting the audibility in the hearing-aid ear from that in the EAS ear. Data from participant P6.

**Figure S1-5. Computing hearing asymmetry.** (A,C) Aided hearing thresholds (black lines) and sound level (colored lines) of the four stimuli in dB HL for the (A) EAS and (C) hearing-aid ear. (B,D) Audibility of each noise band in the (B) EAS ear and (D) hearing-aid ear, with the averaged audibility across frequencies (inset numbers). (E) Hearing asymmetry (∆H) was calculated by subtracting the audibility in the hearing-aid ear from that in the EAS ear. Data from participant P6.

**Figure S1-7. Computing hearing asymmetry**. (A,C) Aided hearing thresholds (black lines) and sound level (colored lines) of the four stimuli in dB HL for the (A) EAS and (C) hearing-aid ear. (B,D) Audibility of each noise band in the (B) EAS ear and (D) hearing-aid ear, with the averaged audibility across frequencies (inset numbers). (E) Hearing asymmetry (∆H) was calculated by subtracting the audibility in the hearing-aid ear from that in the EAS ear. Data from participant P6.

# Sound Localization

Sound localization response of the remaining participants for different listening conditions and sound type (Fig. S2-1 to S2-7.)

***Figure S2-1. Sound localization of participant P6****. Target-response plots are shown for the five listening conditions (rows) and four sound types (columns). Rows correspond to bilateral acoustic only (A – XA), bimodal (A – EX), fully aided (A – EA), cochlear implant only (X - EX), and electro-acoustic only (X – EA). Columns correspond to low-frequency (LF), mid-frequency (MF), mid-to-high frequency (MHF) and high-frequency (HF) sounds. Note that no HF sounds were heard by this participant in the bilateral-acoustic A-XA listening condition (top, right). Colored circles indicate single responses. Colors of inner circles represent the magnitude of localization bias (pEAS) while colors of outer circles represent the magnitude of hearing asymmetry (∆H). Note that the center (0) for pEAS and hearing asymmetry ∆H are color-coded differently for visualization purposes only. Positive ∆H indicates better audibility in the implanted ear.*

***Figure S2-2. Sound localization of participant P6****. Target-response plots are shown for the five listening conditions (rows) and four sound types (columns). Rows correspond to bilateral acoustic only (A – XA), bimodal (A – EX), fully aided (A – EA), cochlear implant only (X - EX), and electro-acoustic only (X – EA). Columns correspond to low-frequency (LF), mid-frequency (MF), mid-to-high frequency (MHF) and high-frequency (HF) sounds. Note that no HF sounds were heard by this participant in the bilateral-acoustic A-XA listening condition (top, right). Colored circles indicate single responses. Colors of inner circles represent the magnitude of localization bias (pEAS) while colors of outer circles represent the magnitude of hearing asymmetry (∆H). Note that the center (0) for pEAS and hearing asymmetry ∆H are color-coded differently for visualization purposes only. Positive ∆H indicates better audibility in the implanted ear.*

***Figure S2-3. Sound localization of participant P6****. Target-response plots are shown for the five listening conditions (rows) and four sound types (columns). Rows correspond to bilateral acoustic only (A – XA), bimodal (A – EX), fully aided (A – EA), cochlear implant only (X - EX), and electro-acoustic only (X – EA). Columns correspond to low-frequency (LF), mid-frequency (MF), mid-to-high frequency (MHF) and high-frequency (HF) sounds. Note that no HF sounds were heard by this participant in the bilateral-acoustic A-XA listening condition (top, right). Colored circles indicate single responses. Colors of inner circles represent the magnitude of localization bias (pEAS) while colors of outer circles represent the magnitude of hearing asymmetry (∆H). Note that the center (0) for pEAS and hearing asymmetry ∆H are color-coded differently for visualization purposes only. Positive ∆H indicates better audibility in the implanted ear.*

***Figure S2-4. Sound localization of participant P6****. Target-response plots are shown for the five listening conditions (rows) and four sound types (columns). Rows correspond to bilateral acoustic only (A – XA), bimodal (A – EX), fully aided (A – EA), cochlear implant only (X - EX), and electro-acoustic only (X – EA). Columns correspond to low-frequency (LF), mid-frequency (MF), mid-to-high frequency (MHF) and high-frequency (HF) sounds. Note that no HF sounds were heard by this participant in the bilateral-acoustic A-XA listening condition (top, right). Colored circles indicate single responses. Colors of inner circles represent the magnitude of localization bias (pEAS) while colors of outer circles represent the magnitude of hearing asymmetry (∆H). Note that the center (0) for pEAS and hearing asymmetry ∆H are color-coded differently for visualization purposes only. Positive ∆H indicates better audibility in the implanted ear.*

***Figure S2-5. Sound localization of participant P6****. Target-response plots are shown for the five listening conditions (rows) and four sound types (columns). Rows correspond to bilateral acoustic only (A – XA), bimodal (A – EX), fully aided (A – EA), cochlear implant only (X - EX), and electro-acoustic only (X – EA). Columns correspond to low-frequency (LF), mid-frequency (MF), mid-to-high frequency (MHF) and high-frequency (HF) sounds. Note that no HF sounds were heard by this participant in the bilateral-acoustic A-XA listening condition (top, right). Colored circles indicate single responses. Colors of inner circles represent the magnitude of localization bias (pEAS) while colors of outer circles represent the magnitude of hearing asymmetry (∆H). Note that the center (0) for pEAS and hearing asymmetry ∆H are color-coded differently for visualization purposes only. Positive ∆H indicates better audibility in the implanted ear.*

***Figure S2-7. Sound localization of participant P6****. Target-response plots are shown for the five listening conditions (rows) and four sound types (columns). Rows correspond to bilateral acoustic only (A – XA), bimodal (A – EX), fully aided (A – EA), cochlear implant only (X - EX), and electro-acoustic only (X – EA). Columns correspond to low-frequency (LF), mid-frequency (MF), mid-to-high frequency (MHF) and high-frequency (HF) sounds. Note that no HF sounds were heard by this participant in the bilateral-acoustic A-XA listening condition (top, right). Colored circles indicate single responses. Colors of inner circles represent the magnitude of localization bias (pEAS) while colors of outer circles represent the magnitude of hearing asymmetry (∆H). Note that the center (0) for pEAS and hearing asymmetry ∆H are color-coded differently for visualization purposes only. Positive ∆H indicates better audibility in the implanted ear.*
